# Responsivity and NEP Improvement of Terahertz Microbolometer by High-Impedance Antenna

**DOI:** 10.3390/s22145107

**Published:** 2022-07-07

**Authors:** Arie Pangesti Aji, Hiroaki Satoh, Catur Apriono, Eko Tjipto Rahardjo, Hiroshi Inokawa

**Affiliations:** 1 Graduate School of Science and Technology, Shizuoka University, Hamamatsu 432-8011, Japan; arie.pangesti.aji.19@shizuoka.ac.jp; 2 Department of Electrical Engineering, Faculty of Engineering, Universitas Indonesia, Depok 16424, Indonesia; catur@eng.ui.ac.id (C.A.); eko@eng.ui.ac.id (E.T.R.); 3 Research Institute of Electronics, Shizuoka University, Hamamatsu 432-8011, Japan; satoh.hiroaki@shizuoka.ac.jp

**Keywords:** terahertz, thermal detector, thermistor, heater, halfwave dipole, folded dipole, antenna, electron beam lithography, responsivity, noise equivalent power

## Abstract

The antenna-coupled microbolometer with suspended titanium heater and thermistor was attractive as a terahertz (THz) detector due to its structural simplicity and low noise levels. In this study, we attempted to improve the responsivity and noise-equivalent power (NEP) of the THz detector by using high-resistance heater stacked on the meander thermistor. A wide range of heater resistances were prepared by changing the heater width and thickness. It was revealed that the electrical responsivity and NEP could be improved by increasing the heater’s resistance. To make the best use of this improvement, a high-impedance folded dipole antenna was introduced, and the optical performance at 1 THz was found to be better than that of the conventional halfwave dipole antenna combined with a low-resistance heater. Both the electrical and optical measurement results indicated that the increase in heater resistance could reduce the thermal conductance in the detector, thus improved the responsivity and NEP even if the thermistor resistance was kept the same.

## 1. Introduction

The terahertz (THz) region originally referred to the spectrum wavelength range from 1000 to 100 μm (0.3 to 3 THz) two decades ago. Then, a broader spectrum overlapping with those from the millimeter wave and far infrared (FIR) regions (0.1 to 10 THz) came to be associated with the THz frequency range because of blurred borders in the transition region from radio electronics to photonics [1,2]. Recently, the improvements in the THz detection and generation methodologies have broadened the THz frequency range further up to 30 THz [3].

In the frequency range around 1 THz, the inter-molecular vibration of prevalent molecules and chemicals formed unique absorption characteristics known as the THz fingerprints. These distinctive features have aroused great interest among many scientists, leading to advances in THz research and development. Numerous THz applications have emerged in the last decade, such as next generation high-speed wireless communication [4,5], nondestructive testing [6], food and water inspection [7], cancer detection in human tissue [8,9], and the remote sensing of ice cloud properties [10].

The major classifications of detectors in the FIR and THz regions are the photon and thermal detectors. For a photon detector, the energy of the incident photon should exceed the energy level difference to generate a carrier, and hence the detector shows selective wavelength characteristics. On the other hand, a thermal detector enables the electrical output by the change in the detector material’s electrical property as the result of the temperature rise by the absorbed incident radiation, and hence its sensitivity is generally wavelength-independent. Photon detectors are usually faster and more sensitive than thermal detectors, but require the use of a cryogenic cooling system in the long wavelength region to suppress the thermal excitation of carriers [11]. A thermal detector is preferable to a photon detector due to the prospective lower operational cost as it can operate at room temperature. However, thermal detectors require an absorber with dimensions sufficiently larger than the wavelength, which should also be thermally isolated to ensure the large temperature rise. The relatively longer wavelength in the THz region makes thermal isolation difficult. Bolometers, pyroelectrics, and thermopiles are among the most used thermal detectors. Some notable methods to improve the moderate responsivity of thermal detectors are the membrane-suspended bolometer structure to reduce thermal conduction [12,13,14,15], a novel THz antenna-coupled bolometer to optimize THz wave collection [16,17], an optical focusing lens [18,19], a metal grating absorber structure [20,21,22], and utilization of a highly sensitive thin film material [23,24]. Further performance enhancement is still widely possible by enabling state-of-the-art fabrication and measurement technologies.

Another class of the THz detector is the detector based on the rectification by electron devices with nonlinear characteristics. Field-effect transistors [25,26], diodes [27,28], and high electron mobility transistors [29,30] are among the most studied rectifying devices for THz detection. They are fast, allow for room temperature operation, and facilitate direct or heterodyne detection of THz signals. However, some challenges exist in their intrinsic cutoff frequency as well as the increasing parasitic effects in the THz frequency, and the performance tends to degrade rapidly with frequency [31]. Therefore, thermal detectors such as bolometers with relatively insensitive performance to the frequency still have some usefulness in the detection of THz waves.

Our group have investigated a novel design of a THz microbolometer by separating the heater and thermistor devices and suspending them above an air cavity [32]. The heater and thermistor devices are electrically separated but thermally connected by an interlayer, allowing performance improvement by independent heater or thermistor optimization process. To identify the incident THz wave reception, an electromagnetic simulation has been performed to investigate an optimum power transfer between the halfwave dipole antenna and heater at the antenna gap [33]. Two important performance metrics of the detector are responsivity, which is defined as the ratio of the output voltage in temperature sensor (thermistor) to the applied input power in the heater [32], and noise equivalent power (NEP), found by measuring the noise generated in the thermistor [34]. As the responsivity and NEP are directly correlated to the detector material, titanium was selected for the heater and thermistor material considering its low thermal and electrical conductivity, resistance to electromigration, and low flicker noise characteristics [35,36,37]. A complex meander thermistor structure has been found to improve the responsivity by four times compared to a straight one [38]. However, resistance increase in a meander thermistor did not improve (reduce) the NEP performance because the heater resistance was not proportionally increased, as revealed by our scaling study [39,40]. Further enhancement by improving heater resistance is necessary to improve the responsivity while keeping the noise voltage fixed. On the other hand, a higher heater resistance also suggests the implementation of the higher antenna impedance for proper power transfer by optimum impedance matching. A folded dipole antenna (FDA), which developed from a standard half-wave dipole antenna with longer effective length, is one of the means to attain a high-impedance antenna. In FDA, the radiating part is miniaturized to a meander structure to reduce the longer effective length of the antenna [41]. It can be implemented on a planar surface and optimized based on its geometrical arrangements. Some studies of FDA have been reported to enhance the high-frequency THz source output power, along with impedance matching technique for antenna-coupled detector applications [42,43,44].

In this study, we proposed a comprehensive study of the implementation of higher heater resistance to improve the responsivity and NEP performance of our microbolometer design. In brief, the contributions of this study are summarized as follows:

1. The proposed bolometer with the reduction in heater width can simultaneously improve the responsivity and NEP performance of the detector.
2. Comprehensive investigation of the microbolometer performance both electrically, by an alternating-current (AC) power supply, and optically, by a THz radiation source.
3. Thermal conduction analysis of the microbolometer structure by a proposed thermal circuit model to verify the electrical and optical measurement results with the average discrepancies of less than 1%.

Finally, the proposed design optimization and the thermal circuit model were successfully fabricated and demonstrated. A performance improvement was attained as well as the method to predict the further design optimization.

## 2. Fabrication Process

**Figure 1:**
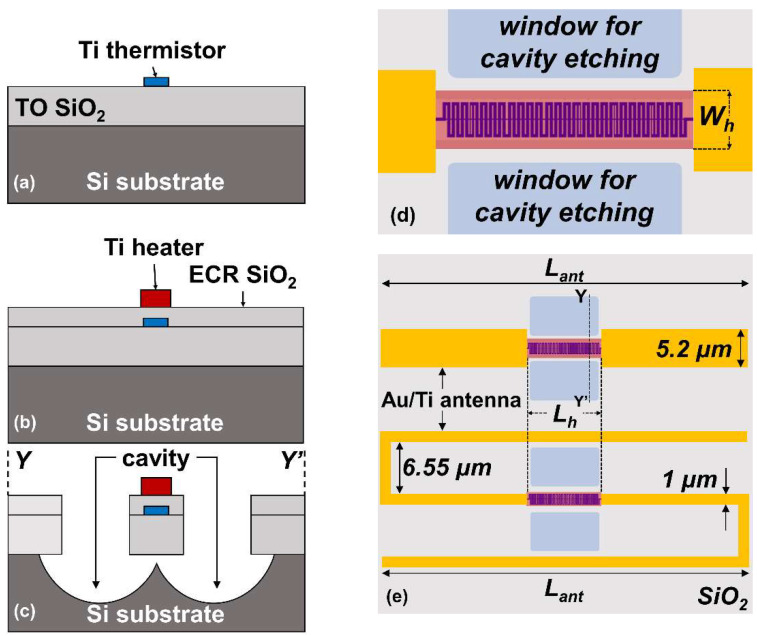
Cross-sectional view of the fabrication processes and top view of the detector: (**a**) 50-nm-thick Ti thermistor deposition on top of 200-nm-thick thermally grown SiO_2_; (**b**) Ti heater deposition on top of 100-nm-thick ECR SiO_2_ interlayer. The heater is aligned on top of the thermistor device for effective thermal coupling; (**c**) cavity hole fabrication for thermal insulation of integrated heater and thermistor; (**d**) heater coupled to the antenna gap on top of meander thermistor. Heater width (*W_h_*) is varied to attain wide resistance range; (**e**) halfwave dipole antenna (top) and folded dipole antenna (bottom) design structure used for THz characterization. Illustrated dimensions are not to scale with the real dimensions.

The studied detector is composed of an integrated titanium (Ti) thermistor, interlayer, Ti heater, and gold (Au) antenna stacked on a high resistivity Si substrate (p-type, *ρ*: 4.2–9.5 kΩ·cm). Fabrication processes were performed sequentially from the bottom to top layer. Prior to thermistor fabrication, 200-nm-thick thermal oxide (TO) SiO_2_ was grown on the Si substrate using the wet oxidation technique under atmospheric pressure for 30 min at 1000 °C, without any pre- and post-treatment. After that, the meander thermistor was patterned and then deposited. Figure 1a illustrates the fabricated thermistor on top of TO SiO_2_. At this point, electrical insulation was then formed by deposition of 100-nm-thick electron cyclotron resonance (ECR) sputtering SiO_2_ interlayer. Once the interlayer as complete, the heater structure was patterned and deposited, as illustrated in Figure 1b. The contact hole for the thermistor feeding line was then patterned and etched on the interlayer by CHF_3_ reactive ion etching (RIE). The Au antenna and measurement pads were patterned and deposited afterwards. More precisely, a 20-nm-thick Ti thin film was first deposited below the Au thin film to strengthen the adherence of the Au film to the interlayer surface. Finally, a deep cavity for thermal isolation was formed by CHF_3_ RIE and SF_6_ plasma etching on the SiO_2_ and Si layers, respectively, as illustrated in Figure 1c. The suspended structure of the thermistor and heater was confirmed by the lateral etching during cavity fabrication. All patterning and metal deposition processes were performed by electron beam lithography (JEOL Ltd., Akishima, Japan, JBX-6300SP) and electron beam evaporation (Shinko Seiki Co., Ltd., Kobe, Japan, SV-A474) system, respectively.

**Figure 2:**
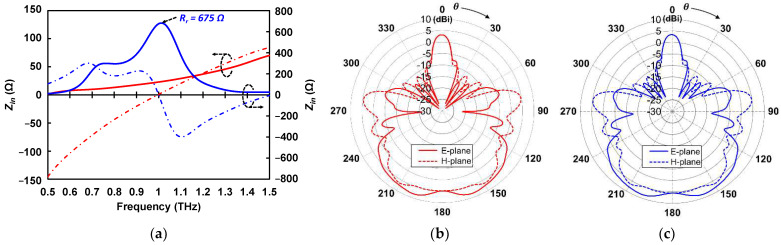
Simulation results of the designed halfwave (red) and folded (red) dipole antenna: (**a**) complex impedance characteristics representing real (solid line) and imaginary (dashed line) parts; (**b**) directivity pattern of halfwave dipole antenna; (**c**) directivity pattern of folded dipole antenna. The direction of *θ* = 180° correspond to the substrate side.

In this study, a common 0.1 μm wide thermistor was designed in meander structure for longer effective length and higher resistance [45]. The heater was designed in fixed length (*L_hea_*) with twelve different widths (*W_h_*) as illustrated in Figure 1d. As we fabricated a wide range of heater resistances, two type of antenna in the shape of halfwave and folded dipole were designed and fabricated with individual antenna length (*L_ant_*) and width (*W_ant_*), as illustrated in Figure 1e. We have simulated the proposed Au antennas on the high-resistivity Si substrate by CST electromagnetic simulator. The antenna length is set so that the imaginary part of the impedance becomes 0 at 1 THz (resonant frequency) as shown in Figure 2a [46]. The proposed folded dipole antenna has an impedance of 675 Ω at 1 THz, while the halfwave dipole antenna has an impedance of 23 Ω at 1 THz. The simulated 1 THz directivity in the broadside (substrate) direction were 4.9 dBi and 5.5 dBi for the halfwave dipole and folded dipole antenna, respectively, as shown in Figure 2b,c. The directivity pattern shown in Figure 2b,c indicates that the folded dipole antenna has a relatively similar radiation pattern to the halfwave dipole antenna.

The use of high-resistivity Si substrate allows the majority of the electromagnetic wave emitted to the substrate direction (θ = 180°). The pattern can be explained by the ratio ε^3/2^ of powers radiated to the substrate and air side, where ε is the permittivity of the substrate [47]. Table 1 summarizes the structure dimension of the thermistor, heater, and antennas.

**Figure 3:**
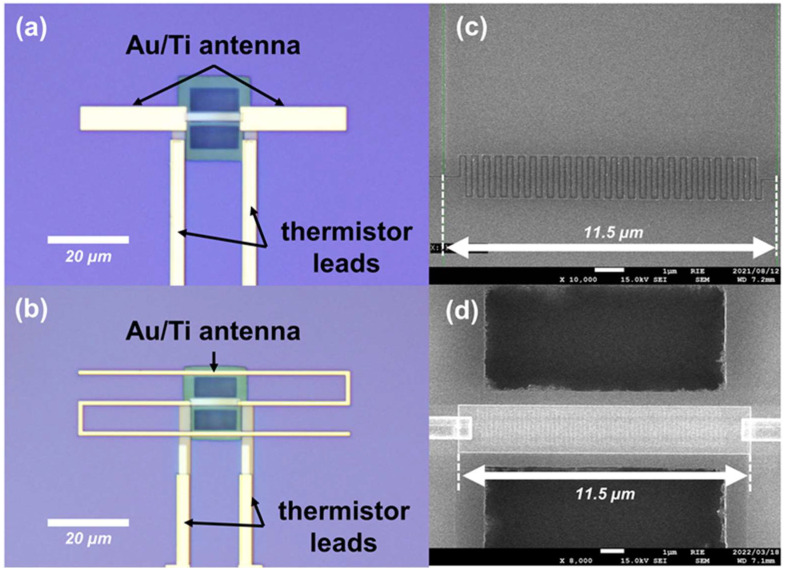
OM and FESEM images of the antenna-coupled detector: (**a**) detector with halfwave dipole antenna; (**b**) detector with folded dipole antenna; (**c**) fabricated meander thermistor on SiO_2_ interlayer with effective length of 89.5 μm; (**d**) suspended heater and thermistor devices above the cavity hole for detector’s thermal isolation from the Si substrate.

Two set of samples were fabricated with the variation in 0.1 and 0.2 μm heater thicknesses. The detector samples were packaged on a chip and wire bonded through the measurement pads for THz optical characterization. While the identical detector samples but without the antenna were also prepared for material parameter and electrical characterization. Figure 3a,b show the structural observation based on optical microscope (OM) for the fabricated detector coupled to halfwave and folded dipole antenna, respectively. Figure 3c,d show the field-emission scanning electron microscope (FE-SEM) observation of the meander thermistor and suspended heater–thermistor structure above the cavity hole, respectively.

## 3. Methods of Characterization

The material parameters of the Ti thermistor and heater were evaluated on a temperature-controlled vacuum prober (Nagase Techno-Engineering, Yokohama, Japan, Grail 21-205-6-LV-R) outfitted with a precision semiconductor parameter analyzer (Agilent, Santa Rosa, CA, USA, 4156C). A standard IV characterization method were used to obtain electrical resistance by the four probe measurements method on the thermistor and heater current and voltage measurement pads. As the resistance changes with the measurement temperature inside the prober, the TCR (*α*), which is defined as the resistance change factor per degree of temperature change, can be calculated by the slope of the linear regression line of resistance against temperature change. Experimentally, TCR was measured by varying the temperature inside the prober from 260 to 300 K in the steps of 10 K. The temperature controller used in our experiment has the accuracy of ±0.25 K to ensure the reliability of the temperature change.

**Figure 4:**
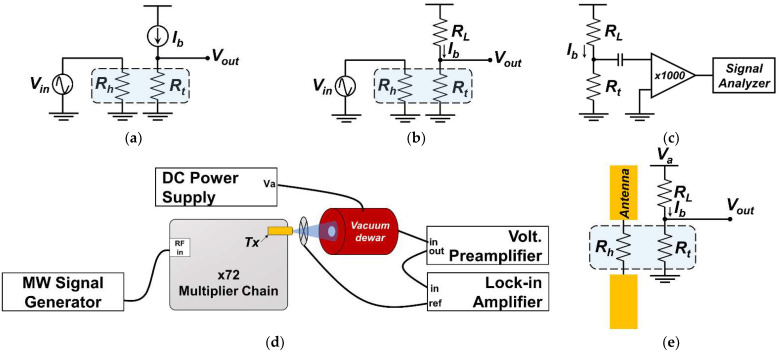
Equivalent circuits and block for detector electrical and optical measurements: (**a**) circuit for electrical responsivity; (**b**) circuit for frequency response; (**c**) circuit for noise analysis; (**d**) THz optical responsivity measurement block; (**e**) electrical connection inside vacuum Dewar.

Electrical responsivity, frequency response, and voltage noise were evaluated at room temperature of 300 K inside the vacuum prober. Since the thermistor is commonly designed for all detector devices, bias current ($I_{b}$) was fixed to 25 μA for all measurements. Responsivity was measured by applying AC electrical power up to 3 μW at a frequency of 10 Hz to the heater and a change in the thermistor voltage output was observed. A constant current source (Yokogawa, Musashino, Japan, GS200) was used to supply the bias current during the responsivity measurement and a lock-in amplifier (Signal Recovery, Oak Ridge, TN, USA, 7270) recorded the voltage output from the thermistor. The circuit diagram for the responsivity measurement is represented in Figure 4a in constant current (CC) mode. The frequency response of the detector was measured electrically on the thermistor by applying amplitude-modulated signal frequency ($f_{m}$) sweep from 1 to 100 kHz to the heater. The second-harmonic thermistor output voltage (2$f_{m}$) was recorded by a lock-in amplifier because the temperature rise is proportional to the square of the temporal amplitude of the modulated signal. Figure 4b shows the circuit diagram for frequency response measurement with external load resistor ($R_{L}$) connected in series with the thermistor. Voltage noise was recorded on the thermistor device over a frequency range of 1 to 100 kHz by an FFT dynamic signal analyzer (Agilent, Santa Rosa, CA, USA, 35670A). Since the thermistor signal amplitude was very low and restricted by the noise produced inside the analyzer, a low-noise and high-gain voltage preamplifier (DL Instruments, Brooktondale, NY, USA, 1201) was used to improve the sensitivity of the spectrum analyzer. The circuit diagram for the noise measurement is depicted in Figure 4c with the addition of an external load resistor ($R_{L}$) connected in series with the thermistor. It is important to note that the external load resistor ($R_{L}$) of 10 kΩ was connected to the thermistor to evaluate frequency response and noise characteristics, and a constant voltage bias source (DL Instruments, Brooktondale, NY, USA, 1211) was used instead of constant current source.

Responsivity to THz radiation was evaluated by illuminating the backside of the detector chip with THz signal generated from a microwave signal generator (Anritsu, Atsugi, Japan, MG3692C) operating at 13.2–14.9 GHz. An amplifier multiplier chain (VDI, Charlottesville, VA, USA, AMC 302) has been used to multiply the microwave frequency by 72 and the signal finally excites the horn antenna (WR1.0 UG-387/UM) at 950–1073 GHz frequency range. A detector chip package was installed inside the vacuum Dewar and arranged 70 mm in front of the transmitter antenna at the center of the radiation beam spot. An optical chopper was placed in between the transmitter ($T_{x}$) antenna and the vacuum Dewar window surface to modulate the source radiation and synchronized to the lock-in-amplifier reference input. Typical power radiated from the source is 0.25 mW. However, we employed a calibrated pyroelectric detector (Spectrum Detector, Lake Oswego, OR, USA, SPH-62-THz), with detector area of 20 mm^2^ to record the radiation intensity at the same place with the detector plane. The effect of water and air absorption at around 1 THz frequency was taken into consideration in the pyroelectric detector calibration process [48]. The incoming THz radiation from the source had a linear polarization and the E-field component of the detector’s antenna was set in parallel to that of the Tx antenna. A bias current of 25 μA was supplied by the DC power supply (Yokogawa, Musashino, Japan, GS200). A voltage preamplifier was used to improve the sensitivity of the lock-in amplifier due to a low output signal intensity generated from the thermistor. Figure 4d shows the experimental block for THz measurement. Embedded inside the vacuum Dewar is a 100 kΩ load resistor ($R_{L}$) connected in series to the thermistor ($R_{t}$) measurement pin as illustrated by the circuit diagram in Figure 4e. No additional optical mirrors nor focusing mechanism were used during measurements.

## 4. Results and Discussion

### 4.1. Electrical Characterization

#### 4.1.1. Material Parameters

**Figure 5:**
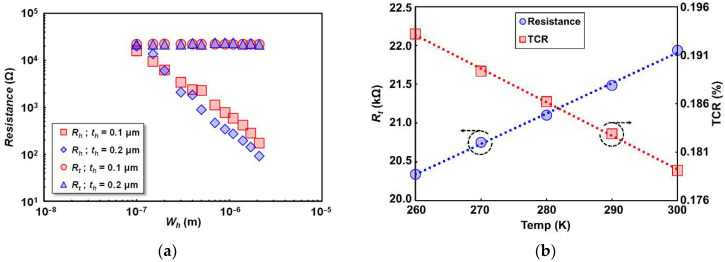
Material parameters of thermistor and heater: (**a**) thermistor ($R_{t}$) and heater ($R_{h}$) resistance dependence on heater width ($W_{h}$) with different heater thickness ($t_{h}$); (**b**) thermistor resistance and TCR dependence on temperature from 240 K to 300 K.

The maximum input current applied to the thermistor and heater were appropriately limited based on the resistance increase of up to 3% to avoid overheating and breaking the devices. A fixed amount of input current was applied to measure thermistor resistance, while the current to heater devices with different widths were set proportionally to the heater widths to obtain constant voltage across them. Resistance at the room temperature ($R_{0}$) of an individual device was taken as the intercept of square input current ($I^{2}$) against resistance increase. Figure 5a shows the electrical resistance of the heater ($R_{h}$) and thermistor ($R_{t}$) in multiple heater design widths ($W_{h}$) on 0.1 and 0.2 μm heater thicknesses ($t_{h}$). $R_{h}$ are linearly decreased with the increase of $W_{h}$ as generally expected from the relation between cross-sectional area of the wire and resistance. A slightly lower $R_{h}$ are shown in the devices with $t_{h}$ of 0.2 μm due to larger cross-sectional area. However, as observed in narrower heater devices, some fluctuations of $R_{h}$ are visible that could be affected by the interlayer surface roughness above the meander thermistor. As for thermistor devices, $R_{t}$ fluctuate insignificantly across different $W_{h}$ and $t_{h}$, without any drastic change.

The temperature coefficient of resistance (TCR) of temperature sensor (thermistor) is an important material property for a detector as responsivity is proportional to it. The TCR (α) is given by $\left(\Delta R/\Delta T\right)/R_{0}$, where ΔR/ΔT is the slope of linear regression line of resistance change per unit temperature, and $R_{0}$ is thermistor resistance at room temperature. Figure 5b shows the relationship of $R_{t}$ and TCR against temperature change from 260 K to 300 K with 10 K steps. It is apparent that resistance increases while TCR is slightly reduced with the temperature. The calculated thermistor resistivity and TCR in room temperature were 1.22 × 10^−6^ Ω·m and 0.179%, respectively, based on the design width of 0.1 μm. That there is no drastic TCR change within different $R_{h}$ revealed that the thermistor electrical parameter is hardly affected by the presence of different heater resistances above it.

#### 4.1.2. Electrical Responsivity

Prior to electrical responsivity measurement, we performed a thermal parameter analysis of the detector. The thermistor, as the detector temperature sensor, is the most important part in the microbolometer structure of our study. Performance measurements were mainly relied on the output given by the thermistor. Hence it is convenient to analyze the thermal resistance of the thermistor to predict the detector performance. As the bias current passes through the thermistor, a uniform self-heating reaction in the thermistor leads to the resistance and temperature increase, thus output voltage increases due to stimulated heater input power can be obtained. Therefore, the thermal property of the thermistor represents the detector performance as it also proportional to the responsivity.

**Figure 6:**
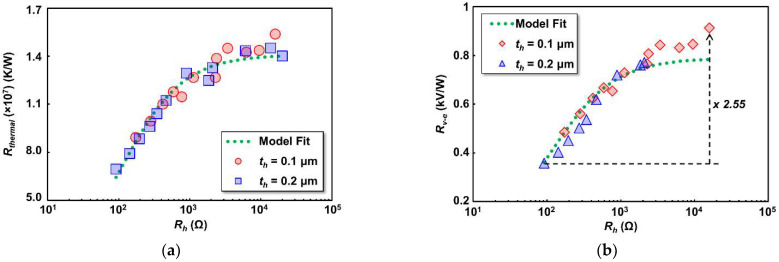
(**a**) Measured thermal resistance ($R_{therm}$) of the detectors with different heater resistance ($R_{h}$); (**b**) detector’s electrical responsivity ($R_{v-e}$) dependence to heater resistance ($R_{h}$). Standard deviation between measured and fitting results are 5.43 × 10^5^ K/W and 48.4 V/W for $R_{therm}$ and $R_{v-e}$, respectively.

**Figure 7:**
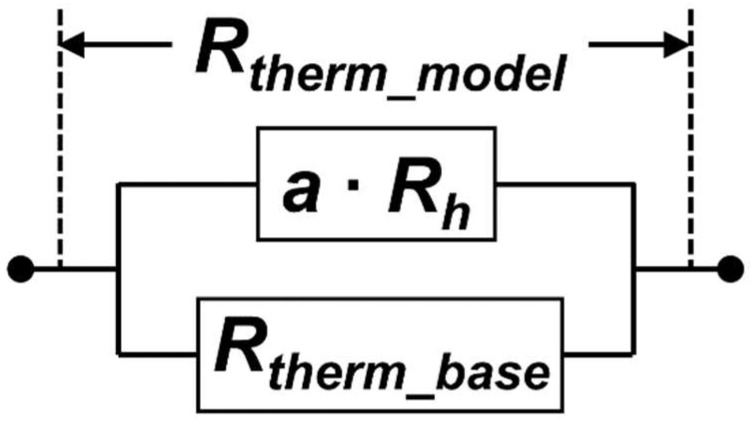
Thermal resistance model in the microbolometer: *a*∙*R_h_* represents the contribution of thermal resistance from the heater (red square box in Figure 1c), and *R_therm_base_* represents the contribution of thermal resistance from other detector structure, i.e., the thermistor (blue square box in Figure 1c) and SiO_2_.

Lateral thermal conductivity (κ) extraction of a metal wire suspended on a Si substrate has been analyzed [49]. It was calculated based on the average electrical resistance ($\bar{R}$) of the wire under bias current (I) expressed by [49]

(1) $$\bar{R}=R_{0}\left[\left(2/mL\right)tan\left(mL/2\right)\right],$$

where $m^{2}=I^{2}R_{0}\alpha /\left(WdL\kappa \right)$. Here, W, d, and L are the width, thickness, and length of the wire, respectively. Equation (1) can be further derived by the Taylor expansion of m with respect to $I^{2}$ into the following expression

(2) $$\bar{R}⋍R_{0}\left(1+\frac{1}{12}\alpha R_{0}R_{therm}I^{2}\right),$$

where $R_{therm}=L/\left(Wd\kappa \right)$ correspond to the thermal resistance of the wire. Therefore, thermal resistance ($R_{therm}$) calculation of our meander thermistor can be simplified as [50]

(3) $$R_{therm}=\frac{12}{R_{0}{}^{2}\alpha }\left(\frac{dR}{dI^{2}}\right),\;$$

where $\left(dR/dI^{2}\right)$ is the slope of the thermistor resistance change for a given square input current. Figure 6a shows the $R_{therm}$ of thermistors calculated by Equation (3) with respect to the change in heater resistance ($R_{h}$) on 0.1 μm and 0.2 μm heater thicknesses ($t_{h}$). As shown, $R_{therm}$ of the thermistor increases as heater resistance increase. As we use a common thermistor design with the same electrical parameter, the main contribution to $R_{therm}$ increase came from the improvement of $dR/dI^{2}$ in the thermistor. It is also revealed that the change in $R_{h}$ could affect to the temperature increase in the thermistor under the same bias current. However, as is also observed, the $R_{therm}$ trend is not linear to the $R_{h}$ increase, and a saturated $R_{therm}$ is predictable as the $R_{h}$ becomes very large. At that point, the thermal parameter of the detector was dominated by another structure, such as the SiO_2_ interlayer or thermistor. Further analysis of the thermal parameter has been made by a fitting line based on a resistor-equivalent parallel circuit of thermal resistance contribution from the heater ($R_{h}$) and another detector structure (i.e., SiO_2_ and thermistor, hereby symbolized as $R_{therm\_base}$), as illustrated in Figure 7. We assume the heater electrical resistance conversion ratio to its thermal counterpart to be a variable of *a*. The model used to predict the calculated thermal resistance ($R_{therm\_model}$) is then given by

(4) $$R_{therm\_model}=\frac{R_{therm\_base}\times \left(a\times R_{h}\right)}{R_{therm\_base}+\left(a\times R_{h}\right)}.$$

The extracted *a* variable is 1.29 × 10^5^ KW^−1^Ω^−1^. This variable can be explained theoretically by the Wiedemann–Franz law that relates the ratio of electrical (σ) to thermal (κ) conductivity parameters of a material (σ/κ = 1/L/T, where L = Lorenz number (2.44 × 10^−8^ WΩ/K^2^) and T = temperature) [51]. Given the experimental temperature of 300 K, the electrical to thermal conductivity ratio is 1.37 × 10^5^ KW^−1^Ω^−^^1^, which is close to the extracted *a* variable value. As shown in Figure 6a, the calculated $R_{therm\_model}$ fit to the measured thermal resistance. This suggests the accuracy of the proposed analytical model for the investigation of thermal contribution in the detector for further performance estimation. As for whether the extracted $R_{therm\_base}$ variable value includes the contribution from the SiO_2_ and thermistor, further data analysis is required to split the contribution of individual components, but this is beyond the scope of the present report.

The responsivity of the detector is defined as the ability of the thermistor to generate voltage output ($V_{out}$) signals with respect to the applied electrical input power ($P_{in}$). In our detector structure, the thermistor is electrically separated but thermally connected to the heater by an SiO_2_ insulation layer. Input power that applied to the heater will increase $R_{h}$ and their average temperature due to Joule heating, hence the heat (power) displaced from the heater stimulates the voltage output increase in the thermistor under bias current ($I_{b}$). The responsivity is proportional to the thermistor material parameters such as resistance and TCR as well as the applied bias current. Miniaturization of the thermistor by a meander structure had the impact of longer effective length and thus increased the resistance with the same lateral length as the stacked heater structure above the thermistor. It is worth recalling that the gap between the antenna’s arm is limited to 11.5 μm. Our group had established that a narrower thermistor reduces TCR but increases resistivity [52]. For the present design, we focus on one thermistor design width of 0.1 μm. However, increase of the $R_{h}$ could also lead to an improvement in responsivity as expected from our previous scaling study. Input power to the heater was given by raising the AC electrical input voltage up to 50 mV at 10 Hz. Thus, the power generated by the heater ($P_{in}$) can be calculated as $V_{hea}{}^{2}/R_{hea}$, where $V_{hea}$ is the voltage output generated from the heater by the heater input–output voltage ratio. A constant $I_{b}$ of 25 μA was applied to the thermistor regardless the $R_{h}$ values. The detector’s electrical responsivity is then expressed as $R_{v-e}=V_{out}/P_{in}$. As $P_{in}$ increase, the thermistor $V_{out}$ will also increase. Therefore, the slope of $V_{out}$ against $P_{in}$ gives the $R_{v-e}$. Figure 6b shows the $R_{v-e}$ variation with different $R_{h}$. Maximum $R_{v-e}$ increase of 2.55 times is observed as the $R_{h}$ increases up to 16 kΩ. The $R_{v-e}$ enhancement, however, is nearly proportional to the extracted detector’s thermal resistance in Figure 6a. It revealed that thermal resistance measured on the thermistor is proportional to the detector’s responsivity. The maximum $R_{v-e}$ is 913 V/W from the device with *R_h_* of 16 kΩ. The calculated responsivity was also given as a fitting model described in Equation (5), with a proportionality constant of *b* is added in series to represent the conversion factor of temperature rise to the output voltage [53]. The analytic model for the electrical responsivity ($R_{v\_model}$) is then given by

(5) $$R_{v\_model}=R_{therm\_model}\times b.\;$$

As can be seen in Figure 6a, the calculated responsivity by the proposed model fit to the measurement results, suggesting the effectiveness of the model in Equation (5) to predict the responsivity trend. The extracted *b* variable is 5.6 × 10^−^^5^ V/K and can be explained by the theoretical extended responsivity analysis from the product of applied bias current ($I_{b}$), extracted TCR (α), thermistor resistance ($R_{t}$), and some additional factor that close to the 1/12 based on equation (2). This additional factor came from temperature rise in a meander thermistor that mainly concentrated at the center part and not in the entire thermistor structure. One concern about the $R_{v-e}$ results in Figure 6b is that three heater devices with the heater width of 0.1, 0.15, and 0.2 μm and thickness of 0.2 μm were defected and unable to be measured. However, their performance can be predicted based on the close proportionality of Figure 6a,b.

**Figure 8:**
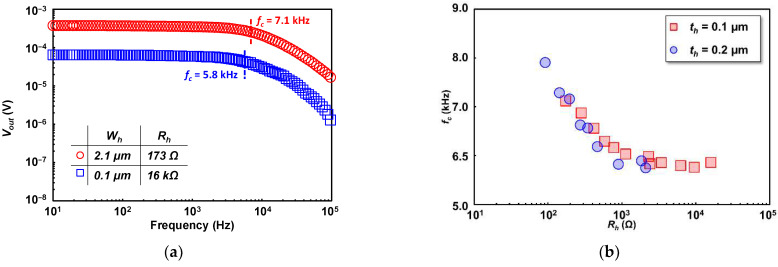
(**a**) Thermistor output voltage to the change of temperature fluctuation frequency, estimated in CC mode; (**b**) detector’s cutoff frequencies ($f_{c}$) dependence to heater resistance.

Figure 8a shows the measured thermistor output voltage ($V_{out}$) response to the change in input AC frequency (f) applied to the heater. Since the load resistor ($R_{L}$) was connected in series with the thermistor ($R_{t}$) according to Figure 4b, the effect of $R_{L}$ has been eliminated by estimating the output voltage in CC mode. The cutoff frequency ($f_{c}$) was used as the fitting parameter to minimize the error between the measured ($V_{o}$) and calculated output voltage $\left|V_{out}\left(f\right)\right|$ by the following fitting equation,

(6) $$\left|V_{out}\left(f\right)\right|=\frac{\left|V_{o}\right|}{\sqrt{1+{\left(\frac{f}{f_{c}}\right)}^{2}}}.\;$$

The $f_{c}$ that gives minimum error extracted as the cutoff frequency. It is notable that higher $R_{h}$ with higher responsivity has smaller $f_{c}$, thus earlier response roll-off is observed. The cutoff frequency ($f_{c}$) dependence to $R_{h}$ is shown in Figure 8b. As shown, $f_{c}$ decreases as $R_{h}$ increases, which is opposite to that of responsivity. This can be explained by the lower thermal diffusivity of the SiO_2_ compared to Ti from the heater [54,55,56]. As the Ti heater width is narrower (resulting in $R_{h}$ increase), the volumetric ratio between Ti and SiO_2_ becomes smaller, and the heat generated in the heater is diffused more slowly. It is also important to note that the extracted $f_{c}$ is based on the temperature fluctuation frequency which is double the AC frequency applied to the heater. Additionally, the measured $f_{c}$ can be further adapted to extract the detector’s thermal response time (τ) by the relation of $½\pi f_{c}$. Indicating that the faster response can be expected for the detector with higher $f_{c}$ as the impact of smaller heat capacitance.

#### 4.1.3. Noise Characteristics

**Figure 9:**
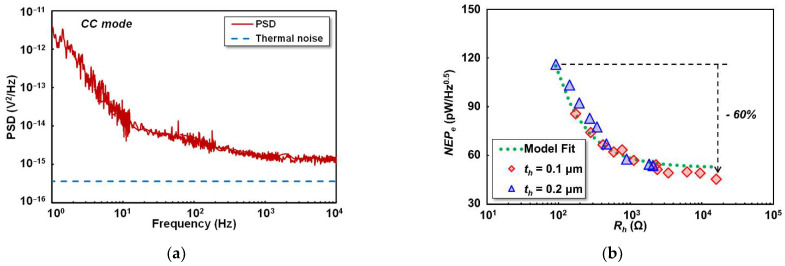
Noise evaluation of the detector: (**a**) power spectrum density (PSD) estimated in CC mode; (**b**) electrical noise equivalent power ($NEP_{e}$) evaluated at 1 kHz, with the standard deviation of 4.5 pW/Hz^0.5^ between measured and fitting results.

Noise equivalent power (NEP) is defined as the input power that gives a signal-to-noise ratio of one for the output noise of 1 Hz and can be expressed as the ratio of voltage noise recorded in the thermistor ($V_{n}$) to the electrical responsivity ($R_{v-e}$). A 10 kΩ external $R_{L}$ was connected in series with the thermistor, as previously shown in Figure 4c. The recorded voltage noise has been converted to the constant current (CC) voltage noise mode thus eliminating the effect of the $R_{L}$. Figure 9a shows the estimated power spectrum density (PSD) in CC mode of the detector under a 25 μA thermistor bias current. The theoretical noise ($V_{n\_therm}$) floor was assumed to be thermal noise generated from $R_{t}$ and given by $\sqrt{4k_{B}TR_{t}}$, where $k_{B}$ is the Boltzmann constant and *T* is the room temperature during measurement. Figure 9b shows the $NEP_{e}$ of the detector against $R_{h}$ change. Assuming the flat response of the detector at the frequency below the $f_{c}$, the NEP was evaluated at 1 kHz with the corresponding voltage noise ($V_{n}$) of 4.15 × 10^−8^ V/Hz^0.5^. The lowest NEP is 45.5 pW/Hz^0.5^ from the device with $R_{h}$ of 16 kΩ. The calculated maximum $NEP_{e}$ reduction is nearly the same as the responsivity improvement due to the common voltage noise in the thermistor. The fitting line for the $NEP_{e}$ results trend was added based on the voltage noise divided by the same model applied for electrical responsivity fitting in Equation (6). As also observed in Figure 9b, the calculated model is fit to the measured $NEP_{e}$.

### 4.2. THz Characterization

Twelve different heater widths were characterized for electrical measurements. However, we used four different heater widths in each heater thickness for THz characterization using two antenna types. The chosen heater widths correspond to the heater resistance near to the antenna’s resonant resistance. Just as the previous electrical characterization, THz optical responsivity is defined as the thermistor voltage output with respect to the input power given by the heater. Instead of direct power supplied to the heater, the THz input power generated from the heater is taken from the capability of the antenna to capture incoming THz radiation from the source to be further transferred into the heater. A good impedance matching between heater and antenna is critical, as one can expect a good power transfer between the two [57]. The output power stated by the THz source equipment datasheet might come to an overestimated value, since not all the incident power falling on the detector’s plane is captured by the antenna. Only a portion of power within the effective antenna area will be absorbed then transferred to the heater load on the antenna gap under good impedance matching. Assuming the THz wave is radiated in uniform power density towards the detector’s plane, the amount of absorbed power by the antenna can be calculated if the effective area of the antenna is determined. Experimentally, we recorded the incident power density ($W_{in}$) by using a calibrated pyroelectric detector. The light receiving area ($A_{e}$) used to calculate input power is assumed as diffraction-limited area based on the square of the effective wavelength on the detector’s surface ($\lambda_{s}{}^{2}$) [58]. The THz optical input power ($P_{in-o}$) is then expressed as $W_{in}\times A_{e}$. Given the relative permittivity of Si substrate of 11.7, the effective wavelength (*λ_s_*) on the detector’s surface is 119 μm. The maximum calibrated *W_in_* within THz source frequency range in detector position was 25 μW/cm^2^. Hence THz optical responsivity can be expressed as $R_{v-o}=V_{out}/\left(W_{in}\times A_{e}\right)$. During optical measurement, we extract the optimum responsivity of each detector devices within the THz source range (950–1073 GHz) where the antennas were designed to have resonant resistance at 1 THz.

**Figure 10:**
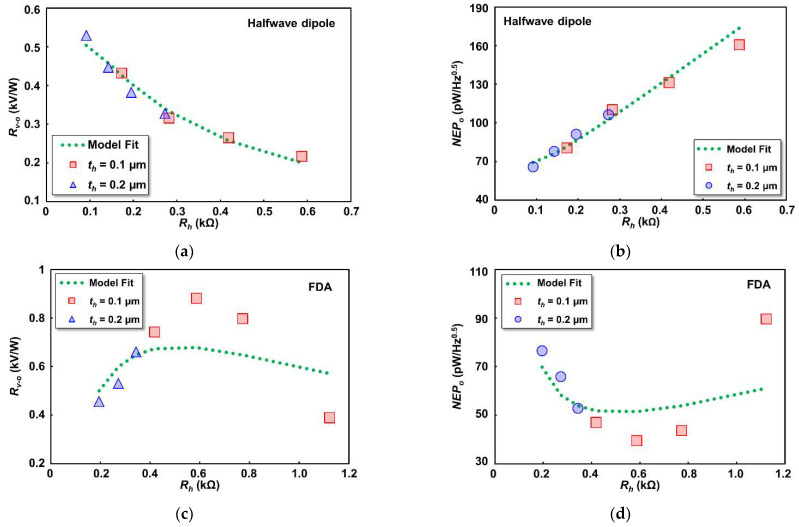
THz optical responsivity ($R_{v-o}$) and NEP dependence on heater resistance for different coupled antenna: (**a**,**b**) halfwave dipole antenna; (**c**,**d**) folded dipole antenna (FDA). The standard deviation of the responsivity and NEP is 16.1 V/W and 5.85 pW/Hz^0.5^ for the halfwave dipole antenna and 124.5 V/W and 13 pW/Hz^0.5^ for the folded dipole antenna, respectively.

The PSD profile for optical NEP ($NEP_{o}$) is taken from the estimated PSD with 100 kΩ load resistor ($R_{L}$) based on the PSD result in electrical measurement ($R_{L}$ = 10 kΩ). The obtained voltage noise at 1 kHz is 3.48 × 10^−08^ V/Hz^0.5^. Figure 10a,b show the $R_{v-o}$ and $NEP_{o}$ trend with the change in $R_{h}$ for the detector coupled to the halfwave dipole antenna. A declining trend is shown for the $R_{v-o}$ results, revealed the ability of the halfwave dipole antenna to transfer the incoming THz power optimally to low resistance heaters. The maximum $R_{v-o}$ and $NEP_{o}$ for halfwave dipole antenna-coupled detector are 530 V/W and 42 pW/Hz^0.5^, respectively, from the device with $R_{h}$ of 91 Ω. Figure 10c,d show the $R_{v-o}$ and $NEP_{o}$ trend with the change in $R_{h}$ for the detector coupled to folded dipole antenna (FDA). The maximum $R_{v-o}$ and $NEP_{o}$ were observed at the heater resistance of 586 Ω, close to the simulated resonant resistance of FDA (675 Ω). The $R_{v-o}$ and $NEP_{o}$ performance worsened as the $R_{h}$ far away from the resonant point. These results indicate that the designed antennas could effectively transfer the incident THz energy to the heater according to their resonant characteristics and matching heater resistance. The maximum $R_{v-o}$ and $NEP_{o}$ for folded dipole antenna-coupled detector is 882 V/W and 39 pW/Hz^0.5^, respectively.

**Figure 11:**
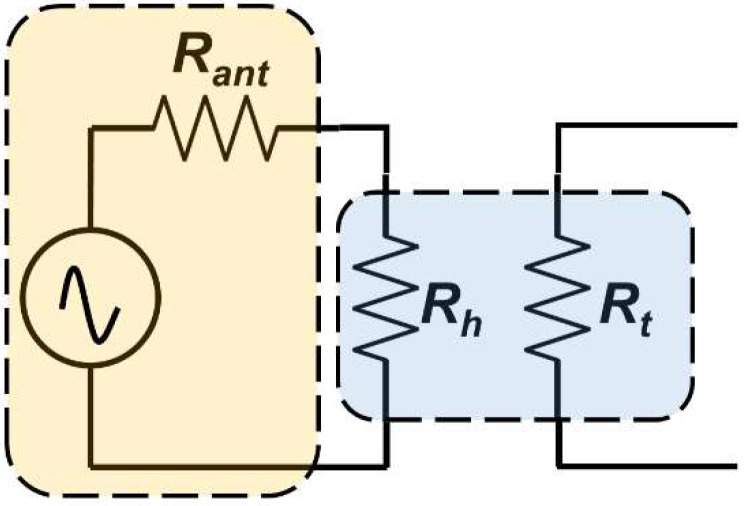
Illustration of circuit diagram for heater input power modelling in THz characterization. Note that the complete model for THz responsivity includes the circuit model in Figure 7.

Further analysis was taken by the model fit line based on the Equation (7). The additional circuit diagram illustrated in Figure 11 is considered due to the fact that input power to the heater was mainly contributed by the antenna, and impedance matching between the antenna and heater significantly affected the THz input power. The input power (power consumption in the heater) can be estimated based on the input voltage ($V_{in}$) generated at the antenna gap and both antenna ($R_{ant}$) and heater ($R_{h}$) resistances. Given the responsivity model in Equation (5) and circuit diagrams in Figure 11 for THz input power, the THz optical responsivity fitting model ($R_{v-o\_model}$) can be summarized by the following equation

(7) $$R_{v-o\_model}=R_{v\_model}\times \frac{4R_{ant}R_{h}}{{\left(R_{ant}+R_{h}\right)}^{2}}\times c.\;$$

The extracted $R_{v-o\_model}$ was obtained by changing the $R_{ant}$ and *c* variable to minimize the discrepancies between measured optical responsivity (*R_v-o_*) and calculated optical responsivity ($R_{v-o\_model}$). Note that extracted *b* variable from Equation (5) together with $R_{therm\_base}$ and *a* variables from Equation (4) are included in Equation (7) because the thermistor design is identical and so the thermal contribution parameters are also the same. The extracted value of $R_{ant}$ for the halfwave dipole antenna device is 20 Ω which is close to the simulated resonant resistance result in Figure 2. Moreover, the extracted value of $R_{ant}$ for FDA is 358 Ω which is far from the simulated resonant resistance of FDA. As for the optical NEP results, fitting trends were taken by dividing the estimated voltage noise in 100 kΩ $R_{L}$ mode with the calculated optical responsivity fitting model in Equation (7). From the graph in Figure 10a,b, we can see the proposed model fit to the THz optical responsivity and NEP for halfwave dipole antenna. Inevitably, there were some higher discrepancies found for fitting the results of FDA in Figure 10c,d due to the limitation of our proposed model for the high-impedance antenna. The extracted $R_{ant}$ from Equation (7) for FDA devices were shifted from the resonant resistance based on the electromagnetic simulation. Nevertheless, the fitting model presented in Figure 10c,d can still be considered for the first order approximation and as a fair comparison to the fitting model of the halfwave dipole antenna. Future works will improve the model for high-impedance antenna coupled to a high-resistance heater to accurately predict the behavior of the device.

The maximum THz responsivity in the present study was found in the heater resistance of 586 Ω, while the electrical measurement results reveal a significant improvement with the higher heater resistance. A higher impedance antenna thus can be expected to further improve the THz responsivity. However, our main intention in this study was to assess the importance of heater resistance increase to the detector’s responsivity, and the effectiveness of the folded dipole antenna with the high resistance heater compared to the classic halfwave dipole antenna. The results highlighted in the present study, nevertheless, also make several noteworthy contributions to further design considerations towards higher detector performance. From the prediction using our proposed equation model, further improvement could be made by reducing the thermal conduction contribution from SiO_2_ by using a thinner interlayer. A careful interlayer fabrication is then needed for a good insulation between the thermistor and heater. A higher impedance antenna could also be used to further improvement in THz responsivity by increasing the number of arms in the folded dipole antenna [59].

In addition to the diffraction-limited area $A_{e}$, the effective area $A_{eff}=D\lambda_{s}{}^{2}/4\pi$ [57] based on the simulated antenna directivity (D) could be used to estimate the input power to the bolometer. Table 2 summarizes the maximum $R_{v-o}$ and minimum $NEP_{o}$ for different assumption of detector areas. The $A_{eff}$ is smaller than $A_{e}$, and thus the $R_{v-o}$ increases and $NEP_{o}$ decreases. Since the $R_{v-o}$ and the $NEP_{o}$ based on the $A_{eff}$ are better than the electrical ones, the former might be overestimated, and the latter might be underestimated. This may be due to the fact that the radiation power is in a wider area than $A_{eff}$ is actually gathered, but some portion of the input power is transmitted and/or reflected, resulting in a reduced detection efficiency. Such an efficiency reduction is not included in the $R_{v-o}$ and $NEP_{o}$ based on the $A_{eff}$.

## 5. Conclusions

This study emphasizes the effect of increasing the heater resistance on the performance of a THz antenna-coupled microbolometer. Devices with various heater widths and thicknesses, as well as antenna types, have been fabricated and studied. The electrical measurement results showed a simultaneous performance improvement in responsivity and NEP by a factor of 2.5 resulting from the combination of a 0.1-μm-wide straight heater and 0.1-μm-wide meander thermistor. Performance comparison between high- and low-resistance heaters for THz wave detection has been made by using a high-impedance antenna and a conventional halfwave dipole antenna, respectively. It was revealed that the responsivity could be improved by matching the impedance of antenna and heater load. Moreover, our simple model revealed that enhancement in responsivity was primarily caused by the increase in the thermal resistance inside the detector. However, as the heater resistance increased, performance improvement became saturated, suggesting the thermal conduction in the detector is dominated by a part other than the heater. Future improvement could be made by minimizing the contribution of the thermistor and/or the interlayer dielectrics to the thermal conduction.

## Figures and Tables

**Table 1:** Device parameters and dimensions.

Device	Material	Dimensions (μm)		
		Length	Width	Thickness
Thermistor	Ti	89.5 ^†^	0.1	0.05
Heater	Ti	11.5 $\left(L_{h}\right)$	0.1–2.1 (*W_h_*)	0.1, 0.2
Halfwave dipole	Au/Ti	52 $\left(L_{ant}\right)$	5.2	0.2/0.02
Folded dipole	Au/Ti	61 $\left(L_{ant}\right)$	1	0.2/0.02

^†^ Effective length.

**Table 2:** Comparison of optical responsivity and NEP for different assumption of detector areas.

Antenna Type	Assumed Area for Input Power Calculation (m^2^)		Max. $R_{v-o}$ (V/W)	Min. NEP_o_ (W/Hz^0.5^)
Halfwave Dipole	$A_{e}=\lambda_{s}{}^{2}$	1.42 × 10^−8^	529.5	65.8
Folded Dipole			881.7	39.5
Halfwave Dipole	$A_{eff}=D\lambda_{s}{}^{2}/4\pi$	3.49 × 10^−9^	2153	16.2
Folded Dipole		4.00 × 10^−9^	3122	11.2

## Data Availability

The data that support the findings of this study are available from the corresponding author (H.I.) upon reasonable request.
